# Seroprevalence and factors associated with hepatitis B virus infection among the hill tribe youths, northern Thailand

**DOI:** 10.1186/s12879-019-3747-3

**Published:** 2019-02-06

**Authors:** Tawatchai Apidechkul

**Affiliations:** 1 Center of Excellence for the Hill tribe Health Research, Mae Fah Laung University, 333 Mo.1 Tasud Subdistrict, Muang District, Chiang Rai Province 57100 Thailand; 2 School of Health Science, Mae Fah Luang University, Chiang Rai, Thailand

**Keywords:** Hepatitis B, Seroprevalence, Hill tribe, Youths, HBsAg, Anti-HBs, Total anti-HBc

## Abstract

**Background:**

Hepatitis B virus (HBV) infection is a major viral infection, particularly in people living in the Western Pacific region, including the hill tribe people living in northern Thailand. This study aimed to estimate the prevalence of HBV infection and to detect the factors associated with HBV infection among hill tribe youths in Thailand.

**Methods:**

A cross-sectional study was conducted to estimate the prevalence and determine the factors associated with HBV infection among hill tribe youths living in northern Thailand. A validated questionnaire and 5 mL blood sample were used for data collection. The Wondfo Diagnostic Kit®, the Wondfo One Step HBsAg Serum/Plasma Test®, and the Wondfo One Step HBsAg Serum/Plasma Test® were used for anti-HBsAg, HBsAg, and total anti-HBc detections, respectively. Logistic regression was used to detect associations between variables with an α = 0.05 significance level.

**Results:**

A total of 836 participants were included in the study; 62.7% were female, 58.9% were aged 15–17 years, 58.7% were Buddhist, 78.4% graduated high school, and 89.1% had no income. The majority were Akha (30.0%), Yao (16.3%), and Hmong (15.8%); 13.2% smoked, 21.5% used alcohol, 13.3% had tattoos, 3.9% experienced drug injection from illegal practitioners, and 35.7% had no history of HBV immunization. The prevalence of HBsAg was 3.0%; anti-HBs, 10.2%; and total anti-HBc, 8.1%. In the multivariate analysis, four variables were found to be significantly associated with HBV infection among the hill tribe youths: age, tribe, work experience, and number of partners. Those aged 18–20 years and 21–24 years had 2.13 times (95%CI = 1.35–3.29) and 2.39 times (95%CI = 1.05–3.90) greater odds of HBV infection, respectively, than those aged 15–17 years. Akha, Lahu, and Hmong youths had 3.12 times (95%CI = 1.07–9.12), 3.71 times (95%CI = 1.21–11.41), and 3.84 times (95%CI = 1.26–11.69) greater odds of HBV infection, respectively, than Lisu youths. Those who had experience working outside of the village had a 1.77 times (95%CI = 1.18–2.98) greater chance of HBV infection than those who did not have experience working outside of the village, and those who had ≥2 partners had a 2.66 times (95%CI = 1.96–3.87) greater chance of HBV infection than those who had no partner.

**Conclusions:**

Effective HBV prevention programs should be promoted in Akha, Lahu, and Hmong youth populations, particularly to those who have sexual partners, work outside of the village and are aged 18–24 years.

## Background

Hepatitis B infection is an infectious diseases with one of the largest impacts on human health, with approximately 257 million infected people worldwide [1]. The target organ of the infection is the human liver [2]. The infection leads to several health problems, including acute and chronic diseases such as hepatitis, cirrhosis, and hepatocellular carcinoma (HCC), etc. [1]. Approximately 80–90% of infections occur in the first year of life, and 30–50% of infections that occur before the age of 6 progress to the chronic stage [3]. The chronic stage of HBV infection eventually leads to the development of HCC during middle age [4, 5]. HCC is one of the most invasive and aggressive cancers [6]. Moreover, HCC rates impact the national health system and health economics of a country.

According to a World Health Organization (WHO) report, people living in the Western Pacific region are the most vulnerable to HBV infection [1], with a prevalence rate of 6.2%. Regarding impacted persons, one-third of hepatitis B cases are reported from the Western Pacific region [7]. China has been recognized as one of the countries with the highest HBV burden, with a reported prevalence of 5.58% [8]. There are several minority populations living in South China [9]. Many of the so-called hill tribe people have been migrating and settling in northern Thailand since the nineteenth century [10]. Today, there are more than three million hill tribe people living in Thailand, which are classified into six main groups: Akha, Lahu, Hmong, Yao, Karen, and Lisu [11]. They have their own culture and lifestyles, including traditional practices that might be related to HBV infection, such as ear piercing and traditional acupuncture [12, 13]. In 2018, approximately 200,000–300,000 hill tribe people lived in Chiang Rai Province, which is located in the northernmost part of Thailand [11]. Today, the hill tribe people still maintain their own daily lifestyles and traditional practices; however, many villagers, particularly those who are living in Chiang Rai Province, are exposed to several risk factors outside their village through globalization. Chiang Rai Province borders Myanmar in the west, China in the north, and the Republic of Laos in the East.

The hill tribes in Thailand have become vulnerable to HBV infection for various reasons, such as their own traditional practices [11], low socioeconomic status [13], poor access to health care, language barriers [14], distance to health care settings [13], and stigmatization from health care providers [13]. In 2018, there were almost 3.5 million hill tribe people living in Thailand; however, some of them were not registered as Thai citizens [11]. Citizenship allows any individual to obtain a national identification card when they reach the age of 7 under Thai regulation [15]. The ID card is used to access all public services, including health care services and school attendance, free of charge.

Due to their living environment, social context, and socioeconomic status, the hill tribe populations in Thailand are at risk of HBV infection, particularly youths who belong to the third or fourth generations of the hill tribes living in Thailand today. The hill tribe youths are at a stage in their lives where it is very possible to be exposed to people outside their villages during different daily activities. Many hill tribe youths are exposed to people outside their villages due to work or attending school in a large city. Exposure to people and experiences outside the village, including sexual partners, tattoos, alcohol and illegal substances, have made them a new vulnerable population for HBV infection. There is little scientific information available regarding hepatitis B infection among hill tribe youths who are sexually active, eager to explore, and exposed to new experiences outside of their villages. Thus, the study aimed to estimate the prevalence and to determine the factors associated with HBV infection among hill tribe youths in northern Thailand.

## Methods

### Study design

A cross-sectional study was performed collect data from hill tribe youths.

### Study setting

The participants were recruited from 60 selected hill tribe villages. Five villages of each tribe were selected by a simple random method from lists of the hill tribe villages located in Chiang Rai Province, northern Thailand. In 2016, there were 749 hill tribe villages in Chiang Rai, which included 316 Lahu villages, 243 Akha villages, 63 Yao villages, 56 Hmong villages, 36 Karen villages, and 35 Lisu villages. In 2016, a total of 41,366 hill tribe families lived in Chiang Rai Province [16].

### Study population

The study population comprised hill tribe youths aged 15–24 years.

### Eligible population

Eligible populations were hill tribe youths from one of the six hill tribes living in Chiang Rai Province.

### Inclusion and exclusion criteria

The inclusion criteria were as follows: a) the participant self-identified as a member of one of the six hill tribes, b) the participant was aged between 15 and 24 years at the date of data collection, and c) the participant was fluent in Thai. However, selected participants who had a physical or mental condition that led to the inability to provide essential information regarding the study protocols were excluded from the study.

### Sample size

The sample size was calculated based on a formula for calculating the sample size in a cross-sectional study [17];

$$ \mathrm{n}=\left[{{\mathrm{Z}}^2}_{\upalpha /2}\mathrm{PQ}\right]/{\mathrm{e}}^2 $$

where n = sample size required, Z = 1.96, *P* = 0.10 [18], Q = 0.90 and e = 0.05. An average of 138 cases per tribe was required. Based on six tribes, a total of 829 participants were required for the analysis.

### Research instruments

A questionnaire and 5 mL blood specimens were used as research instruments. A questionnaire was developed from a literature review and consultations with experts in the field. The questionnaire consisted of 31 questions categorized into three sections. In the first section, 10 questions were used to collect general information from the participants, such as sex, age, tribe, and marital status. In the second section, 8 questions were used to collect information on risk behaviors such as smoking, alcohol drinking, tattoos, and ear piercing. In the last section, 13 questions were used to collect information on the sexual behaviors of the participants, such as the number of partners, use of condoms during sexual intercourse, and sexual orientation.

### Research instrument development

The questionnaire was tested for validity by the item-objective congruence technique (IOC), which was performed by three external experts in the relevant fields, a virologist, an infectious disease specialist, and an infectious epidemiologist. Questions with a score of less than 0.50 were excluded from the questionnaire, questions with a score between 0.50–0.70 were revised before use, and questions with a score of > 0.70 were used without modification.

Afterward, the questionnaire was piloted on 20 persons (10 males and 10 females) in the Mae Chan district of Chiang Rai Province who were similar to the study participants, with the aim of assessing the reliability and feasibility of the questionnaire.

### Process of data gathering

After identifying all sixty selected hill tribe villages, village headmen were contacted 2 days before data collection. Access to villages was granted by district government officers. The lists of the target subjects were obtained from the village headmen. In each village, all eligible subjects were informed about the research objectives and protocols by the village headman. The study samples were randomly selected from the lists provided. All selected samples were appointed in the village. Upon reaching the village, all selected samples were provided all essential information and written informed consent was obtained before completing the questionnaire. Five milliliter blood samples were voluntarily collected by a professionally licensed medical technologist. Blood samples were kept in a proper container with cold packs and transferred to the laboratory on the same day.

### Laboratory method

Laboratory tests were performed at the Mae Fah Luang Medical Laboratory. A rapid immunochromatographic method was used to detect anti-HBs and HBsAg markers. The Wondfo Diagnostic Kit® was used to detect ant-HBsAg with 97.3% sensitivity and 99.2% specificity. The Wondfo One Step HBsAg Serum/Plasma Test® was used to detect HBsAg with 96.2% sensitivity and 99.3% specificity. For the anti-HBc, the Wondfo One Step HBsAg Serum/Plasma Test® was used with 93.0% sensitivity and 99.0% specificity.

#### Laboratory interpretation

Participants who were negative for HBsAg, total anti-HBc, and anti-HBs were susceptible to HBV infection. Participants who were negative for both HBsAg and total anti-HBc but positive for anti-HBs were defined as having immunity due to hepatitis B vaccination. Participants who were negative for HBsAg but positive for total anti-HBc and anti-HBs were defined as having immunity due to natural infection. HBV infection was defined as people who were positive for HBsAg and/or total anti-HBc after excluding individuals who had a serological profile of previous vaccination against HBV [19, 20].

### Statistical analysis

All questionnaires and laboratory information were coded and double entered into an Excel sheet. Data were checked for errors, including missing values, before analysis. Data were analyzed by SPSS version 24, 2016 (SPSS, Chicago, IL). Descriptive and inferential statistics were used for analysis; general characteristics of participants were described by means, standard deviations, and percentages. Logistic regression was used to detect the associations between independent variables (general characteristics, risk behaviors, and sexual behaviors) and the dependent variable, HBV infection, at the α = 0.05 significance level. The “ENTER” method was chosen in the logistic regression model, and statistically nonsignificant variables were excluded from the model; therefore, only statistically significant variables were included in the interpretation.

## Results

A total of 836 participants were recruited for the study, and nobody refused to participate. The majority of participants were females aged 15–17 years (mean = 17.5, SD = 2.4) and of the Akha tribe. Most participants were single, in school, had no income, and had 4–6 family members. Half of the participants were Buddhist (Table 1).

**Table 1:** General characteristics of the participants

Characteristics	n (%)
Total	836 (100.0%)
Sex	
Male	312 (37.3)
Female	524 (62.7)
Age (years)	
15–17	492 (58.9)
18–20	248 (29.7)
21–24	96 (11.5)
Tribe	
Akha	251 (30.0)
Lahu	110 (13.2)
Hmong	132 (15.8)
Yao	136 (16.3)
Karen	130 (15.5)
Lisu	77 (9.2)
Marital status	
Single	791 (94.6)
Other	45 (5.4)
Family members (persons)	
≤ 3	92 (11.0)
4–6	529 (63.3)
7–10	181 (21.7)
≥ 11	34 (4.1)
Religion	
Buddhism	491 (58.7)
Christianity	339 (40.6)
Islam	6 (0.7)
Education	
Illiterate	21 (2.5)
Primary school	31 (3.7)
High school	655 (78.4)
Vocational school	129 (15.4)
Occupation	
Unemployed	62 (7.4)
Student	685 (81.9)
Farmer	22 (2.6)
Laborer	67 (8.1)
Income	
No	745 (89.1)
Yes	91 (10.9)
Living place	
Own house	590 (70.6)
Dormitory	208 (24.9)
Other	38 (4.5)

Regarding the risk behaviors, different kinds of drug use were detected among the participants: 13.2% smoked, 21.5% drank alcohol, 2.3% used methamphetamines, and 4.3% used marijuana. Two-thirds experienced ear piercing, and 13.3% were tattooed. Some participants reported blood transfusions (3.9%), organ transplants (1.0%), a history of drug injection from illegal practitioners (3.9%), and acupuncture (1.8%). Only 8.5% were immunized for HBV, and 2.9% had at least one family member who had hepatitis. One-third of the participants reported that they had a sexual experience, 44.9% did not use a condom during their first sexual intercourse, and 11.5% had ≥2 partners (Table 2).

**Table 2:** Risk behaviors among the participants

Characteristics	n	%
Smoker		
No	726	86.8
Yes	110	13.2
Alcohol use		
No	656	78.5
Yes	180	21.5
Methamphetamine us		
No	817	97.7
Yes	19	2.3
Heroin use		
No	823	98.4
Yes	13	1.6
Crystal methamphetamine use		
No	825	98.7
Yes	11	1.3
Opium use		
No	824	98.6
Yes	12	1.4
Marijuana use		
No	800	95.7
Yes	36	4.3
Tattooed		
No	725	86.7
Yes	111	13.3
Ear piercing		
No	302	36.1
Yes	534	63.9
History of blood transfusion		
No	803	96.1
Yes	33	3.9
History of organ transplant		
No	828	99.0
Yes	8	1.0
History of medical surgery		
No	733	87.7
Yes	103	12.3
Injection from illegal practitioners		
No	803	96.1
Yes	33	3.9
Acupuncture		
No	821	98.2
Yes	15	1.8
Work experience outside the village		
No	693	82.9
Yes	143	17.1
Work experience abroad		
No	826	98.8
Yes	10	1.2
Used a shared toothbrush		
No	656	78.5
Yes	180	21.5
History of hepatitis B vaccination		
Yes	71	8.5
No	298	35.7
Not sure	261	31.2
Unknown	206	24.6
Family member history of hepatitis		
No	481	57.5
Yes	24	2.9
Not sure	120	14.4
Unknown	211	25.2
Sexual experience		
No	593	70.9
Yes	243	29.1
Used a condom during first sexual intercourse		
No	109	44.9
Yes	120	49.4
Unremembered	14	5.7
Number of partners (persons)		
None	593	70.9
1	147	17.6
≥ 2	96	11.5

The prevalence rate of HBsAg positivity was 3.0%, anti-HBs positivity was 10.2%, total anti-HBc positivity was 8.1%, and both anti-HBs and anti-HBc positivity was 0.92%. The rate of negativity for anti-HBs but positivity for total anti-HBc was 7.4%. The prevalence rate of HBV infection was 10.3% (Table 3).

**Table 3:** Prevalence of HBsAg and anti-HBs

Characteristics	n	%
Total	836	100.0
HBsAg		
Negative	811	97.0
Positive	25	3.0
Received HBV vaccination	3	12.0
No history of HBV vaccination	6	24.0
Not sure	9	36.0
Unknown	7	28.0
Anti-HBs		
Negative	751	89.8
Positive	85	10.2
Received HBV vaccination	3	3.5
No history of HBV vaccination	32	37.6
Not sure	31	36.5
Unknown	19	22.4
Total anti-HBc		
Negative	768	91.9
Positive	68	8.1
Received HBV vaccination	0	0.0
No history of HBV vaccination	12	17.6
Not sure	27	39.8
Unknown	29	42.6
Anti-HBs-positive and anti-HBc-positive	4	0.9
Anti-HBs-negative and anti-HBc-positive	64	7.4
HBV infection	86	10.3

In the univariate analysis, five variables were associated with HBV infection among the hill tribe youths: age, tribe, marital status, work experience outside the village, and number of sexual partners. However, in the multivariate analysis, four variables remained associated with HBV infection. Those aged 18–20 years and 21–24 years had 2.13 times (95%CI = 1.35–3.29) and 2.39 times (95%CI = 1.05–3.90) greater odds of HBV infection, respectively, than those aged 15–17 years. Akha, Lahu, and Hmong youths had 3.12 times (95%CI = 1.07–9.12), 3.71 times (95%CI = 1.21–11.41), and 3.84 times (95%CI = 1.26–11.69) greater odds of HBV infection, respectively, than Lisu youths. Those who had experience working outside the village had a 1.77 times (95%CI = 1.18–2.98) greater chance of HBV infection than those who did not work outside the village, and those who had ≥2 sexual partners had a 2.66 times (95%CI = 1.96–3.87) greater chance of HBV infection than those who did not have a sexual partner (Table 4).

**Table 4:** Univariate and multivariate analyses of factors associated with HBV infection

Factors	n	%	OR	95%CI	*p*-value	OR_Adj_	95%CI	p-value
Sex								
Male	312	37.3	1					
Female	524	62.7	0.70	0.47–1.06	0.707			
Age (years)								
15–17	492	58.9	1			1		
18–20	248	29.7	2.14	1.38–3.34	0.001^a^	2.13	1.35-3.29	0.001^a^
21–24	96	11.4	2.39	1.33–4.30	0.004^a^	2.39	1.05-3.90	0.005^a^
Tribe								
Akha	251	30.0	3.05	1.05–8.87	0.040^a^	3.12	1.07-9.12	0.037^a^
Lahu	110	13.2	4.30	1.41–13.10	0.010^a^	3.71	1.21-11.41	0.022^a^
Hmong	132	15.8	3.65	1.20–11.02	0.022^a^	3.84	1.26-11.69	0.018^a^
Yao	136	16.3	2.09	0.66–6.60	0.207.	2.46	0.77–7.87	0.127
Karen	130	15.5	2.02	0.63–6.45	0.232	1.86	0.58–5.98	0.293
Lisu	77	9.2	1			1		
Marital status								
Single	791	94.6	1					
Other	45	5.4	2.26	1.11–4.60	0.025^a^			
Number of family members (persons)								
1–3	92	11.0	1					
4–6	529	63.3	1.40	0.67–2.92	0.361			
7–10	181	21.7	1.47	0.65–3.31	0.343			
≥ 11	34	4.1	1.97	0.64–6.04	0.233			
Religion								
Buddhism	491	58.7	1					
Christianity	339	40.6	0.81	0.53–1.24	0.345			
Islam	6	0.7	1.22	0.14–10.62	0.855			
Education								
Illiterate	21	2.5	1					
Primary school	31	3.7	2.77	0.51–14.90	0.235			
High school	655	78.4	1.36	0.31–5.94	0.651			
Vocational school	129	15.4	1.64	0.35–7.62	0.527			
Occupation								
Unemployed	62	7.4	1					
Student	685	81.9	0.83	0.39–1.75	0.632			
Farmer	22	2.6	2.20	0.68–7.14	0.186			
Labor	67	8.1	1.03	0.39–2.73	0.948			
Income								
No	745	89.1	1					
Yes	91	10.9	1.03	0.52–1.90	0.993			
Smoker								
No	726	86.8	1					
Yes	110	13.2	1.34	0.77–2.33	0.287			
Alcohol use								
No	656	78.5	1					
Yes	180	21.5	1.36	0.86–2.16	0.187			
Methamphetamine use								
No	817	97.7	1					
Yes	19	2.3	1.24	0.35–4.34	0.732			
Heroin use								
No	823	98.4	1					
Yes	13	1.6	1.20	0.26–5.50	0.811			
Crystal methamphetamine use								
No	825	98.7	1					
Yes	11	1.3	0.65	0.08–5.18	0.690			
Tattooed								
No	725	86.7	1					
Yes	111	13.3	1.13	0.63–2.00	0.674			
Ear piercing								
No	302	36.1	1					
Yes	534	63.9	0.94	0.62–1.43	0.788			
History of blood transfusion								
No	803	96.1	1					
Yes	33	3.9	0.65	0.19–2.16	0.484			
History of medical surgery								
No	733	87.7	1					
Yes	103	12.3	1.04	0.57–1.90	0.889			
Injection from illegal practitioners								
No	803	96.1	1					
Yes	33	3.9	0.65	0.19–2.16	0.484			
Acupuncture								
No	821	98.2	1					
Yes	15	1.8	1.01	0.22–4.56	0.984			
Used a shared toothbrush								
No	656	78.5	1					
Yes	180	21.5	1.02	0.62–1.65	0.937			
History of hepatitis B vaccination								
Yes	71	8.5	1					
No	298	35.7	1.58	0.64–3.90	0.319			
Not sure	261	31.2	1.96	0.79–4.83	0.143			
Unknown	206	24.6	1.56	0.61–3.97	0.346			
Family history of hepatitis								
No	481	57.5	1					
Yes	24	2.9	0.58	0.13–2.53	0.471			
Not sure	120	14.4	1.05	0.59–1.87	0.852			
Unknown	211	25.2	0.89	0.55–1.46	0.670			
Work experience outside the village								
No	693	82.9	1					
Yes	143	17.1	1.92	1.20–3.07	0.006^a^	1.77	1.18-2.98	0.004^a^
Work experience abroad								
No	826	98.8	1					
Yes	10	1.2	2.88	0.73–11.30	0.130			
Sexual experience								
Yes	243	29.1	1.10	0.71–1.71	0.648			
No	593	70.9	1					
Used a condom during first sexual intercourse								
Yes	120	49.4	1					
No	109	44.9	1.49	0.70–3.17	0.293			
Does not remember	14	5.7	1.26	0.25–6.23	0.775			
Number of sexual partners (person)								
None	593	84.7	1			1		
1	67	9.6	0.43	0.15–1.22	0.113	0.57	0.28–1.19	0.099
≥ 2	40	5.7	2.97	1.90–4.30	0.033^a^	2.66	1.96-3.87	0.047^a^

^a^ Significant at α = 0.05

## Discussion

In this study, the prevalence of HBsAg was 3.0%, the prevalence of anti-HBV was 10.2%, and the prevalence of HBV infection was 10.3%. The hill tribe youths in Thailand who were of the Akha, Lahu, and Hmong tribes; were older; had more than one partner; and had experienced working outside the village had a higher risk of HBV infection.

Thailand has included the HBV vaccine in their expanded program of immunization (EPI) since 1992 [21–23]. All children are immunized with at least three doses of the HBV vaccine, with the first dose given 24 h after birth. In 2017, after 25 years of HBV vaccination through the EPI program, a 99.0% HBV vaccine coverage was reported for the whole country [22]. However, in our study, only 71 out of 836 participants (8.5%) confirmed having received HBV vaccination; 31.2% reported that they were not sure if they had been immunized, and 24.6% reported that they did not know their HBV immunization status. However, there were only 3 cases positive for HBsAg and 3 cases positive for anti-HBs among those who had a history of HBV immunization. These data differ from the 90–95% efficacy rate of the HBV vaccine after 3 standard doses declared by the Ministry of Public Health in Thailand [24]. This reflects the level of access to health care services among the hill tribe people in Thailand, which was reportedly low due to factors such as distance, language and stigmatization [14, 25]. There are also many factors influencing the efficacy of HBV immunization, such as nutritional status [26], route of vaccine delivery [27], age at vaccination, underlying diseases [28, 29], and host genetics [30]. Moreover, with low parental education rates and low health literacy, including the administration of the HBV immunization in children while they are young, may have led to missing responses to question in the study which might impact the analysis. During the data collection, many sources of information were investigated regarding the HBV immunization status of a participant. A study conducted at the Thai-Myanmar border reported that administering HBV vaccines to people living in the border area was highly effective in preventing the disease [31].

Regarding HBsAg seroprevalence, countries in the Western Pacific region had a rate of 6.1% [1]. In Taiwan, the seroprevalence of anti-HBsAg and total anti-HBc was 8.1% and increased according to age, particularly in those aged 18–25 years [32]. In 2015, Leroi et al. [17] reported that the overall seroprevalence of HBsAg in the Thai general population was 0.6–3.1%. Lana, et al. [21] reported that the HBs-carrier rate of Thai people aged 11–20 was 0.69%. These findings are lower than the seroprevalence detected in our study among hill tribe youths in Thailand.

Moreover, Banks, et al. [33] reported that the hepatitis B seroprevalence among pregnant women who lived on the Thailand-Myanmar border was 8.3%. Another study that was conducted with children and adolescents with HIV infection in six countries in Southeast Asia, including Thailand, reported that the prevalence of HBsAg was 4.7% [34]. Pichainarong, et al. [35] reported that the hepatitis B seroprevalence among married hill tribe women in northern Thailand was 8.2%. These studies presented HBsAg seroprevalence rates close to the rates in our study due to similar population demographics. Another reason might be an increase in the number of people who are exposed to people outside their village (17.1%). Today, many hill tribe people prefer to work outside of their village to earn money to support their family [36]. In fact, hill tribe people, particularly those aged below 25 years, should have low HBsAg since they were born after the implementation of HBV vaccination in Thailand. Another reason for high anti-HBsAg and anti-HBc levels among hill tribe people is due to low seroclearance, particularly in those who were infected at an older age [37, 38].

Regarding age and HBV infection, Taleban et al. [39] reported that increased age was one of the most significant factors for HBV infection in Iran. A study among health care professionals in Ethiopia in 2017 also reported that people who were older in age had a significantly greater risk of HBV infection than those younger in age [40]. Another study conducted among prisoners in Iran reported that age was the significant factor associated with HBV infection [41]. These findings support our study, which found that the older age group had a greater risk of HBV infection among the hill tribe youths in northern Thailand, even though they were born after the national implementation of HBV vaccination in Thailand.

Slovakia reported that some group of people had a greater risk than others; the Roma population had a greater risk of HBV infection than both the non-Roma population and those with tattoos [42]. Our study also found that some tribes had a greater risk of HBV infection than others.

The number of partners was highly associated with HBV infection in pregnant women in Ethiopia [43] and Tanzania [44]. A study among female workers in Laos also reported that the number of partners was associated with HBV infection [45]. Another study in Bangkok reported that the number of partners was associated with HBV infection [46]. These findings are similar to our study, which found that the number of partners was significantly associated with HBV infection among hill tribe youths, particularly those who had experience working outside of the village. Some studies [47, 48] conducted in hill tribe people in Thailand reported that working outside of the village was a factor associated with HIV and hepatitis B coinfection in northern Thailand.

Marital status was found to be associated with HBV infection in the univariate model; however, there was no statistical association with HBV infection in the multivariate model. A possible reason is for this result may be that many hill tribes have a monogamous culture, except the Akha who accept polygamy in their culture [25]. Those who have multiple partners often do so before marriage; once they are married, the opportunity for HBV infection is reduced. Several studies have also shown that marital status is not associated with HBV infection [49, 50].

Recall bias and misclassification were major limitations in the study. Recall bias [51, 52] happened since many questions regarding previous experiences were asked. For instance, a large proportion answered “not sure” or “unknown” when asked about their history of hepatitis B vaccination. These answers may have led to misclassification during analysis. However, the researchers were aware of these issues and repeated the questions during the interview to ensure the participants understood the questions before providing their answers.

All the HBsAg-positive participants were provided recommendations to seek further medical investigation, proper care and treatment. Those who were not immune were provided additional information regarding immunization at a registered hospital to protect against HBV infection.

## Conclusion

The hill tribe youths in Thailand are at risk of hepatitis B infection, particularly those who work outside the village and have multiple sexual partners. Due to globalization, young children in the hill tribes should be immunized with the HBV vaccine through the EPI program of Thailand to ensure that they are immune to HBV infection. Moreover, suitable health education programs, such as safe sex practices, should be developed and implemented in Akha, Lahu, and Hmong youth populations aged ≥18 years.

## Abbreviations

Anti-HBc: Hepatitis core antibody
Anti-HBs: Hepatitis B surface antibody
CI: Confident interval
EPI: Expanded program of immunization
HBsAg: Hepatitis B surface antigen
HBV: Hepatitis B virus
HCC: Hepatocellular carcinoma
IOC: Item-objective congruence technique
MSM: Men who have sex with men
WHO: World Health Organization

## References

[1] World Health Organization (WHO). Hepatitis B: Key facts. http://www.who.int/news-room/fact-sheets/detail/hepatitis-b

[2] World Health Organization (WHO). Guidelines on hepatitis B and C testing. http://www.who.int/hepatitis/publications/hepatitis-b-guidelines/en/.

[3] Centers for Disease Control and Prevention. Viral hepatitis B. https://www.cdc.gov/hepatitis/hbv/index.htm

[4] Kao WY, Su CW, Tan EC, Lee PC, Chen PH, Tang JH, et al. Proton pump inhibitors and risk of hepatocellular carcinoma in patients with chronic hepatitis B or C. Hepatology. 2018. 10.1002/hep.30247. 10.1002/hep.30247 30175498

[5] Choi J, Kim GA, Han S, Lee W, Chun S, Lim YS. Longitudinal assessment of three serum biomarkers to detect very early stage hepatocellular carcinoma. Hepatology. 2018. 10.1002/hep.30233. 10.1002/hep.30233 30153338

[6] Lisa PW, Vrushak D, Nikolaos P (2015). Hepatocellular carcinoma: A comprehensive review. World J Hepatol.

[7] World Health Organization (WHO). Global hepatitis report 2017. http://apps.who.int/iris/bitstream/handle/10665/255016/9789241565455-eng.pdf;jsessionid=699D49FD849542F6768BBDEC9279E810?sequence=18.

[8] Xu C, Chen T (2018). Global prevalence of hepatitis B virus infection and prevention of mother-to-child transmission. Lancet Gastroenterol Hepatol.

[9] Gao X, Wang X, Zhu B. The distribution of Chinese minority populations and its change based on the study of the Hu Huanyong line. Int J Anthropol Ethnol. 2017;1(2). 10.1186/s41257-017-0004-9.

[10] Princess Maha Chakri Siridhorn Anthropology center. Hill tribe. 2018. http://www.sac.or.th/main/index.php

[11] Lukas H. Southeast Asian hill tribes and the opium trade – The historical and social-economic background of the marginalization of minorities using the example of Thailand. Austria: University of Vienna. p. 2017.

[12] Apidechkul T (2016). A 20-year retrospective cohort study of TB infection among the hill tribe HIV/AIDS populations, Thailand. BMC Infectious Disease.

[13] Apidechkul T, Jiamton S, Jareinpituk S, Kaewkungwal J (2007). Sexual behaviors and HIV infection among pregnant hill tribe women in northern Thailand. Southeast Asian J Trop Med Public Health.

[14] Apidechkul T, Laingoen O, Suwannaporn S (2016). Inequity in accessing health care service in Thailand in 2015: a case study of the hill tribe people in Mae Fah Luang district, Chiang Rai, Thailand. J Health Res.

[15] Department of Provincial Administration, Ministry of Interior, Thailand. Act on the card 2011. http://www.moi.go.th/portal/page?_pageid=814,1036627,814_1036665&_dad=portal&_schema= PORTAL.

[16] The hill tribe welfare and development center, Chiang Rai province (2016). Hill tribe population. The hill tribe welfare and development center.

[17] Peter B, Leslie EW, Mark RS, Charles EM (2005). Ethic and sample size. Am J Epidemiol.

[18] Leroi C, Adam P, Khamduang W, Kawilapat S, Neo-Giang-Huong, Ongwandee S, et al. Prevalence of chronic hepatitis B virus infection in Thailand: a systematic review and meta-analysis. In J Infect Dis 2016; 51: 136–43. 10.1016/j.ijid.2016.08.017 27580678

[19] Trepo C, Chan HL, Lok A (2014). Hepatitis B virus infection. Lancet.

[20] Centers for Disease Control and Prevention (CDC). Interpretation of hepatitis B serologic test results. 2018. http://www.cdc.gov/hepatitis/hbv/pdfs/serologicchartv8.pdf.

[21] Lana C, Sigrun R, Rania AT (2018). Status and progress of hepatitis B control through vaccination in the South-East Asia Region, 2992-2015. Vaccine.

[22] World Health organization (WHO). EPI Fact Sheet: Thailand. http://www.searo.who.int/immunization/data/thailand.pdf

[23] Posuwan N, Wanlapakorn N, Sa-Nguanmoo P, Wasitthakasem R, Vichaiwattana P, Klimfueng S (2016). The success of a universal hepatitis B immunization program as part of Thailand’s EPI after 22 years’ implementation. PLoS One.

[24] Ministry of Public Health, Thailand. Efficacy and effectiveness of hepatitis B vaccine among Thai children. https://ddc.moph.go.th/th/site/index

[25] Apidechkul T, Wongnuch P, Sithisarn S, Ruanjai T (2016). Health status of Akha hill tribe in Chiang Rai province, Thailand. J Pub Health Dev.

[26] Scott DP, Inna GO, Gregory AP (2015). The weight of obesity on the human immune response to vaccine. Vaccine.

[27] Anja S, Beate K (2017). Vaccine responses in newborns. Semin Immunopathol.

[28] Teresa A, Jane B, John RB, Bonnie BB, Wilbur HC, Julian H (2018). Report on WHO meeting on immunization in older adults: Geneva, Switzerland, 22-23 march 2017. Vaccine.

[29] Pham TT, Le HM, Nguyen DT, Maertens K, Leuridan E, Theeten H (2018). Assessment of the timely administration of the hepatitis B and BCG birth dose and the primary infant vaccination schedule in 2015-2016 in the Mekong Delta, Vietnam. Vaccine.

[30] Akcay IM, Katrinli S, Ozdil K, Doganay GD, Doggany L (2018). Host genetic factors affecting hepatitis B infection outcomes: insights from genome-wide association studies. World J Gastroenterol.

[31] Angela D, Rebecca H, Aung MN, Mary EG, Moo KP, Joy K (2017). Strategies for the prevention of perinatal hepatitis B transmission in a marginalized population on the Thailand-Myanmar border: a cost-effectiveness analysis. BMC Infect Dis.

[32] Lai MW, Lin TY, Tsao KC, Huang CG, Hsiao MJ, Liang KH (2012). Increased seroprevalence of HBV DNA with mutations in the S gene among individuals greater than 18 years old after complete vaccination. Gastroenterology.

[33] Banks T, Kang J, Watts I, Tyrosvoutis ME, Min AM, Tun NW (2016). High hepatitis B seroprevalence and risk factors for infection in pregnant women on the Thailand-Myanmar border. J Infect Dev Ctries.

[34] Aurpibul L, Kariminia A, Vibol U, Fong MS, Le ON, Hansudewechakul R (2018). Seroprevalence of hepatitis B among HIV-infected children and adolescents receiving antiretroviral therapy in the TREAT Asia pediatric HIV observational database. Pediatr Infect Dis J.

[35] Pichainarong N, Chaveepojnkamjorn W, Luksamijarulkul P, Sujirarat D, Apidechkul T (2003). Hepatitis B carrier among married hill tribe women in northern Thailand. Southeast Asian J Trop Med Public Health..

[36] Apidechkul T (2018). Prevalence and factors associated with type 2diabetes mellitus and hypertension among the hill tribe elderly populations in northern Thailand. BMC Public Health.

[37] Teerapat U, Tassanee S, Pattama K, Pitchayachuda C, Kamonwan S, Gaidganok S (2017). Role of quantitative hepatitis B surface antigen in predicting inactive carriers and HBsAg seroclearance in HBeAg-negative chronic hepatitis B patients. Medicine (Baltimore).

[38] Catherine MN, John SL (2014). Natural history of chronic hepatitis B: phases in a complex relationship. World J Gastroenterol.

[39] Teleban R, Moafi M, Ataei B, Yaran M, Nokhodian Z, Kassaian N, et al. Seroprevalence of hepatitis B infection and associated risk factors among drug users in drop-in centers of Isfahan, Iran. Int J Prev Med. 2018;9(46) DOI: 10.4103. 10.4103/ijpvm.IJPVM_382_16 PMC5981222 29899884

[40] Demsiss W, Seil A, Fiseha T (2018). Hepatitis B and C: seroprevalence, knowledge, practice and associated factors among medicine and health science students in Northeast Ethiopia. PLoS One.

[41] Moradi G, Gouya MM, Azimizan ZF, Mohamadi BA, Darvishi S, Aghasadeghi MR (2018). Prevalence and risk factors for HBV and HCV in prisoners in Iran; a national bio-behavioral surveillance survey in 2015. Tropical Med Int Health.

[42] Drazilova S, Janicko M, Kristian P, Schreter I, Halanova M, Urbancikova I, et al. Prevalence and risk factors of hepatitis B virus infection in Roma and non-Roma people in Slovakia. Int J Environ Res Public Health. 2018;15(5). 10.2290/ijerph15051047. 10.3390/ijerph15051047 PMC5982086 29789486

[43] Amsalu A, Ferede G, Eshetie S, Tadewos A, Assegu D. Prevalence, infectivity, and associated risk factors on hepatitis B virus among pregnant women in Yirgalem hospital, Ethiopia: implication of screening to control mother-to-child transmission. J Pregnancy. 2018:8435910. 10.1155/2018/8435910. 10.1155/2018/8435910 PMC6098924 30174956

[44] Kapinga D, Aboud S (2018). Seroprevalence and factors associated with hepatitis B virus infection in pregnant women attending antenatal clinic in Karagwe district council, Tanzania. Int J Infect Dis.

[45] Xaydalasouk K, Strobel M, Buisson Y, Black AP, Muller CP (2018). Seroprevalence and risk factors of hepatitis B and C virus infections in female workers of Lao garment factories. PLoS One.

[46] Linkins RW, Chonwattana W, Holtz TH, Wasinrapee P, Chaikummao S, Varangrat A (2013). Hepatitis A and hepatitis B infection prevalence and associated risk factors in men who have sex with men, Bangkok, 2006-2008. J Med Virol.

[47] Apidechkul T, Pongwiriyakul S (2016). Factors associated with HIV and HBV co-infection in northern Thailand. Asian Pac J Trop Dis.

[48] Apidechkul T (2016). Epidemiology of the hill tribe HIV/AIDS populations, Thailand. J Med Assoc Thail.

[49] Jobayer M, Chowdhury SS, Shamsuzzaman SM, Islam MS (2016). Prevalence of hepatitis B virus, hepatitis C virus, and HIV in overseas job seekers of Bagladesh with the possible routes of transmission. Mymensigngh Med J.

[50] Bakarey AS, Akinboade IO, Aken’Ova YA (2018). Transmission transmissible hepatitis B virus markers of infection among sickle cell disease patients receiving care at a tertiary health facility in Ibadan, southwest Nigeria. J Immunoassay Immunochem.

[51] Coughlin SS (1990). Recall bias in epidemiological studies. J Cli Epidemiol.

[52] Belbasis L, Bellou V (1793). Introduction to epidemiological studies. Methods Mol Biol.

